# Systemic and medullary blastic plasmacytoid dendritic cell invasion

**DOI:** 10.1002/jha2.533

**Published:** 2022-08-23

**Authors:** Chea Mathias, Brun Sophie, Loyens Maxime

**Affiliations:** ^1^ Nimes Caremeau Hospital Montpellier University Montpellier France

**Keywords:** blast, dendritic cells, leukaemia, neoplasm

1

A 67‐year‐old man with history of idiopathic thrombocytopenia since 2010 was referred by his primary care physician for leukocytosis (98,000/mm^3^) with thrombocytopenia (<5000 mm^3^) and anemia (Haemoglobin at 7,6 g/dl) to the emergency department.

For 1 month, the patient has lost 1 kg and presented axillary adenopathies and a chronic cough.

Workup performed at hospital showed leukocytosis at 128,000/mm^3^, anemia at 7,1 g/dl, thrombocytopenia at 15 000/mm^3^ with 91% circulating lymphoma‐like cells composed of small cells with moderate basophilic and agranular cytoplasm (panel C; Giemsa stain, objective ×100).

Surprisingly, bone marrow smear showed a massive invasion (95%) by a population of medium‐sized blast cells, some of which are microvacuolated and elongated by a cytoplasmic pseudopod (tadpole or “hand mirror” appearance, Figure 1, panel A Giemsa stain, 50× objective and panel B Giemsa stain, 100× objective). Cytology was suggestive of a blastic plasmacytoid dendritic cell neoplasm (BPDCN). Interestingly, blasts lose their pseudopods when they pass in bloodstream in our case.

**FIGURE 1:**
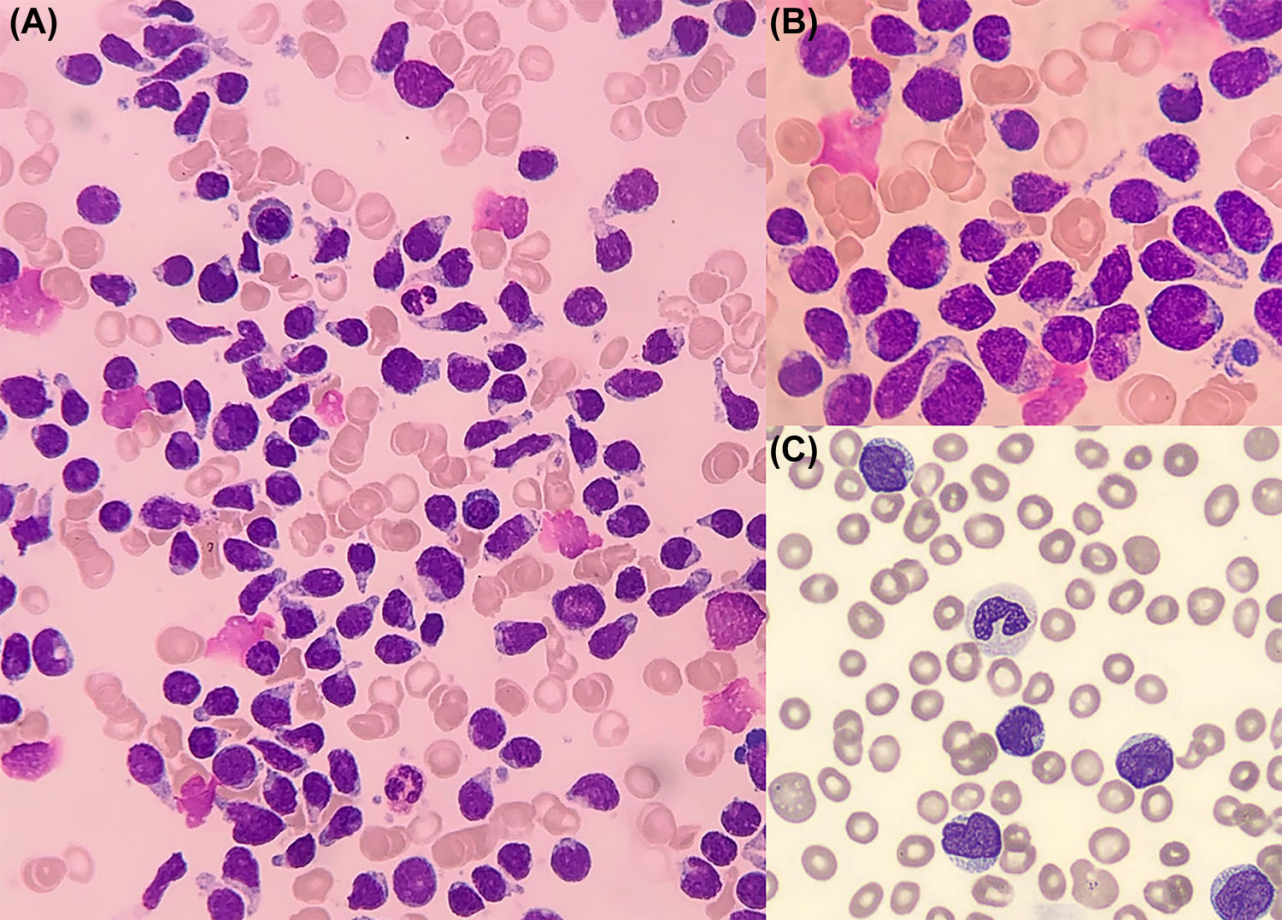
Giemsa stain (A): blastic plasmacytoid cells on bone marrow smear, 50× objective, (B) blastic plasmacytoid cells on bone marrow smear, 100x objective, (C) blastic plasmacytoid cells on peripheral blood smear, 100× objective

Immunophenotyping on CD45+ low cells revealed that they were positive for CD4, CD56, HLA‐DR, CD123, CD304 (low), cTCL1, BadLamp, FcER1, NG2 (low), ILT3, CD36, CD38, and CD2 confirming the diagnosis of BPDCN (LpDC score at 3/5). Cytogenetic analysis and next generation sequencing showed no specific mutation but a complexe karyotype (44∼45,XY,der(4)t(4;5)(q35:q?14),5,?del(7)(q36),‐11,del(?12)(p11),‐13,?+21,+mar[8]/46,XY[12]).

A few days after admission to the hospital, the patient died of septic shock complicated by multivisceral failure.

BPDCN is a very rare (less than 1% of acute leukemia) and aggressive disease with very poor outcome but therapeutic options are evolving every day. This hemopathy mainly affects elderly men (median age of 70 years), and whose initial skin involvement is almost constant except in our case.

Herein, we describe an atypical case of BPDCN with no skin damage but a massive systemic infiltration with lymphoid‐like cells mimicking a lymphoma bloodstream dissemination contrasting with a typical BPDCN cells medullary invasion.

## CONFLICT OF INTEREST

The authors declare that there is no conflict of interest that could be perceived as prejudicing the impartiality of the research reported.

## FUNDING INFORMATION

The authors received no specific funding for this work.

## DATA AVAIBILITY STATEMENT

The data that support the findings of this study are available from the corresponding author upon reasonable request.

## ETHICS STATEMENT

No research on human was performed on this study.

